# Factors that impact Australian early career nurses' intentions to remain in their position and the profession: A prospective cohort study

**DOI:** 10.1111/jonm.13803

**Published:** 2022-10-07

**Authors:** Amanda Cottle‐Quinn, Marion Tower, Rob Eley

**Affiliations:** ^1^ School of Nursing, Midwifery and Social Work The University of Queensland Brisbane QLD Australia; ^2^ Griffith University Brisbane QLD Australia; ^3^ The University of Queensland Brisbane QLD Australia; ^4^ Princess Alexandra Hospital Brisbane QLD Australia

**Keywords:** education, employment, health workforce, nursing, nursing graduate, work engagement

## Abstract

**Aim:**

The aim of this work is to identify factors that impact on early career nurses' intentions to remain in their current position and compare with what impacts on intention to remain in the profession.

**Background:**

Early exits of nurses from a position and the profession are a result of evolving factors. A lack of longitudinal follow‐up impedes knowledge about these factors or what proportion of temporary exits become permanent.

**Method:**

The study used prospective cohort survey design. The sample was obtained from non‐probability convenience sampling of graduating nursing students from two universities. Data was collected across three time points over 12 months, commencing in November 2016.

**Results:**

The professional turnover rate was 6.7% in total. Higher Work Environment, Support and Encouragement scores and Stress in personal life were the only predictors of intending to remain in the profession. Statistical modelling could not predict intention to remain in current position.

**Conclusion:**

There were differences identified when comparing intention to remain in a position and the profession. Nurses are satisfied with their career choice and intend to remain in the profession, although many are intending to move positions.

**Implications for Nursing Management:**

To effectively retain the nursing workforce, stakeholders must focus on the work environment, appropriate support and remuneration, and facilitate career progression.

## 1 BACKGROUND

Graduates in all professions have higher rates of job mobility when compared with those who have spent longer in the workforce (Kidd et al., 2014). This remains true for nursing (Frijters et al., 2007; North et al., 2014), influencing health workforce supply. In Australia, this trend continues (Health Workforce Australia, 2014). This turnover is financially expensive for the industry (Henderson et al., 2015; McCalla‐Graham & De Gagne, 2015). It can also lead to a decrease in the quality of treatment through lack of continuity of care (Eley et al., 2007; Huntington et al., 2012).

Intent to leave a position is thought to be related to discouraging and exhausting work experiences (Henderson et al., 2015; McCalla‐Graham & De Gagne, 2015), and low job satisfaction (Bontrager et al., 2016; Kenny et al., 2016). For early career nurses, being married or working in small nonmetropolitan hospital (Cho et al., 2014), or being older or working in a nonpreferred ward (Beecroft et al., 2008), may also increase intention to leave a current position.

Intent to leave the profession may be due to a lack of understanding regarding graduate employment intentions and a reduction of expenditure on health services, including supported graduate nurse employment (Gilmour et al., 2016; Henderson et al., 2015). However, job satisfaction (Gurkova et al., 2013; Walker & Campbell, 2013), and negative perceptions of the work environment (Dechawatanapaisal, 2018; Guerrero et al., 2017) can also play a role. Being younger (Camerino et al., 2008; Hasselhorn et al., 2005), being more highly qualified, and being male (Hasselhorn et al., 2005) are also predictors of intent to leave the profession.

It can be argued that job mobility in nursing simply reflects the female domination of the profession as younger nurses take on parenting or home duties (Health Workforce Australia, 2014). However, in Australia (Wilkins & Inga, 2018) and internationally (Tourangeau et al., 2017), job mobility because of increased education, career changes, upskilling and moving location are common. Indeed, turnover from one job to another within nursing has been reported to achieve career advancement, better working conditions, develop new skills and meet professional goals, often resulting in superior financial earnings for the individual (Camerino et al., 2008; Cho et al., 2014; Eley et al., 2007; Kidd et al., 2014).

Therefore, the early exits of early career nurses from a position and the profession may be a result of evolving factors. Currently a lack of longitudinal follow‐up means it is not known what proportion of the exits from a position are also from the profession, nor whether they were for career or academic progression, or due to dissatisfaction. Intra‐individual changes are a methodological approach to data collection that is currently missing from the limited knowledge on graduate nurse employment outcomes in Australia. There are clearly differences between what causes early career nurses to want to leave a position, compared with the profession. An increased understanding of the causal influences on early career nurses' intent to remain both in the position and the profession should lead to evidence‐based strategies that may result in higher retention rates.

The current paper, reporting on the doctoral work of the first author (Cottle, 2019), addresses the gaps in the literature by identifying the factors that impact on retention in a position, and the profession, using a longitudinal design to track intraindividual changes over time with a focus on intent to remain as opposed to intent to leave.

## 2 METHODS

This paper reports on one research question from a larger study (Cottle, 2019). The other two research questions related to initial employment outcomes and employment settings and the findings have been published previously (Cottle‐Quinn et al., 2021). This study asked: “Which factors do early career nurses attribute their intent to remain in the profession and their current positions?” It was guided by two theoretical frameworks, Stages of Transition Theory (Duchscher, 2008) and Transition Shock (Duchscher, 2009).

### 2.1 Participants

The target population was final‐year nursing students enrolled in undergraduate nursing programmes in Australia. A convenience sample of 583 graduating nursing students was recruited from two universities (A and B). Participants were recruited using three main activities with repetition. First, information about the study was made available on the learning platforms. Second, face‐to‐face presentations were provided by the researcher during teaching time, and finally information was distributed through university‐related social media sites such as Facebook.

### 2.2 Sample size

Sample size in surveys is reliant on level of significance (measured at .05), and power (measured at 80%). In this study, logistic regression analysis of up to 10 factors was proposed. With a required minimum of 10 cases per variable for binary logistic regression, an expected response rate of 50% and a further 25% lost to follow up, the initial response rate of the convenience sample exceeded that required for this analysis.

### 2.3 Research instrument

The study employed an online survey titled *Early Career Nurse Employment Experience Survey*, which included adaptations of the *Casey‐Fink Readiness for Practice Survey*© (Casey et al., 2011) and *Casey‐Fink Nurse Retention* S*urvey©* (revised, 2009) (Buffington et al., 2012). The surveys were adapted for appropriateness to the Australian context and included the following sections:

1. Work environment: measured using an adaptation of the Work Environment, Support and Encouragement scale from the Casey‐Fink Nurse Retention Survey © (revised, 2009).
2. Job satisfaction: measured using the original Casey‐Fink Nurse Retention Survey © (revised, 2009) (Buffington et al., 2012) scale. In the original analysis, each of the 13 items on the scale (rated using Likert scale [1 = *very dissatisfied*, 2 = *moderately dissatisfied*, 3 = *neither satisfied nor dissatisfied*, 4 = *moderately satisfied*, 5 = *very satisfied*]) was reported individually; however, as an exploratory factor and for the purpose of modelling, in this research, all 13 items were taken as a scale, allowing for a continuous variable to be created.
3. Stress: The original Casey‐Fink Nurse Retention Survey © (revised, 2009) one statement was, “I am experiencing stress in my personal life”, asking for self‐report of the level of agreement using a Likert scale (1 = *strongly disagree*, 2 = *disagree*, 3 = *agree*, 4 = *strongly agree*). Given the context of this study and its focus on employment, this item was extended, and a follow‐up question added. Participants were asked to respond to the statement, “I am experiencing stress in my personal life that affects my work.” Participants who scored 3 = *agree* or 4 = *strongly agree* were then asked to identify what was causing their stress and to answer whether this stress had a positive or negative effect on their ability to work using a self‐report Likert scale (1 = *extremely negative*, 2 = *somewhat negative*, 3 = *neither positive nor negative*, 4 = *somewhat positive*, 5 = *extremely positive*).
4. Support: Researcher‐designed questions identifying what employers do to support graduates and what graduates desired to facilitate their transition were asked, including whether participants identified as taking part in a formal structured support programme, its name and length.
5. Intent to remain: Researcher designed questions including “What do you think could be done to improve Registered Nurse retention in the Australian workforce?” In Phases Two and Three, one item that formed part of the Readiness for Practice scale pertained to intention to remain and three items from the Work Environment, Support and Encouragement scale. In Phases Two and Three, employed early career nurses were also asked how many more whole years they intended to remain in the nursing workforce in Australia, and in the final survey only, how many more whole years they intended to remain in their current registered nurse position. Researcher‐designed open‐ended questions, “What keeps you working in your current position?” and “What might cause you to leave your current position?” expanded the quantitative results.

Following face and content validity by an expert panel (*n* = 8) minor language and formatting changes were made.

The *Early Career Nurse Employment Experience Survey* was distributed via Qualtrics (Qualtrics, 2017). Recruitment and Phase One was in October 2016 as participants were completing their degrees; Phase Two approximately 6 months later April/May 2017, with Phase Three occurring in November/December 2017.

### 2.4 Ethical considerations

Ethical clearance was obtained from the UQ Behavioural & Social Sciences Ethical Review Committee (BSSERC), clearance number: NMSW 2016/02.

### 2.5 Data analysis

IBM SPSS Statistics version 24 was used to analyse the data using Cronbach's alpha coefficient, binary logistic and multiple regression, and the Pearson product–moment correlation coefficient. To review data across commonly accepted categories, some continuous variables were collapsed into categorical variables. Specifically related to this paper.

- Intent to remain in position: Collapsed to binary >7.5 years and ≤7.5 years as the average job tenure is 7.5 years for full‐time Australian workers (Wilkins & Inga, 2018)
- Intent to remain in profession: Collapsed to binary >10 years and ≤10 years as the average career tenure is 10 years in Australia (Fell, 2018).

The results are described using the format described by Pallant (2010). The final sample retained in Phase Three (*n* = 194) was used for analysis as this provided a complete set of data for each of the participants. Missing values were treated as suggested by Pallant (2010) with cases excluded analysis by analysis. Responses to open ended questions were coded, classified into categories, and counted, with a higher number suggesting a more prominent category.

## 3 RESULTS

### 3.1 Description of sample

Response rate in Phase One was 50.3% (*n* = 293). The final sample retained from Phase Three was 194 with overall loss to follow‐up of 33.8% (*n* = 99). The study sample was predominately female (89.2%), average age 26, with 63% under 25 (for full demographics table, see Cottle‐Quinn et al., 2021).

### 3.2 Employment outcomes

**FIGURE 1:**
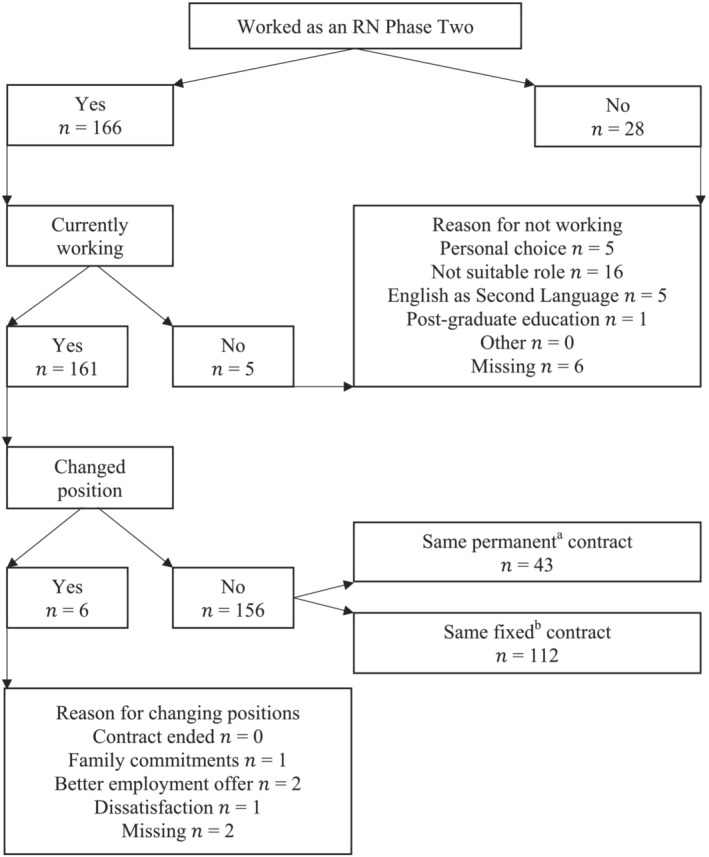
Flow of participants' employment outcomes at Phase Two. *Note*: ^a^An employee employed on a permanent contract is employed on an on‐going basis and is entitled to paid leave including annual leave and sick and carer's leave, and is usually entitled to written notice when their employment ends, or payment instead of notice (Australian Government, 2022). ^b^Fixed term contract employees are also entitled to paid leave including annual leave and sick and carer's leave but are employed for a specific period of time or task (Australian Government, 2022).

**FIGURE 2:**
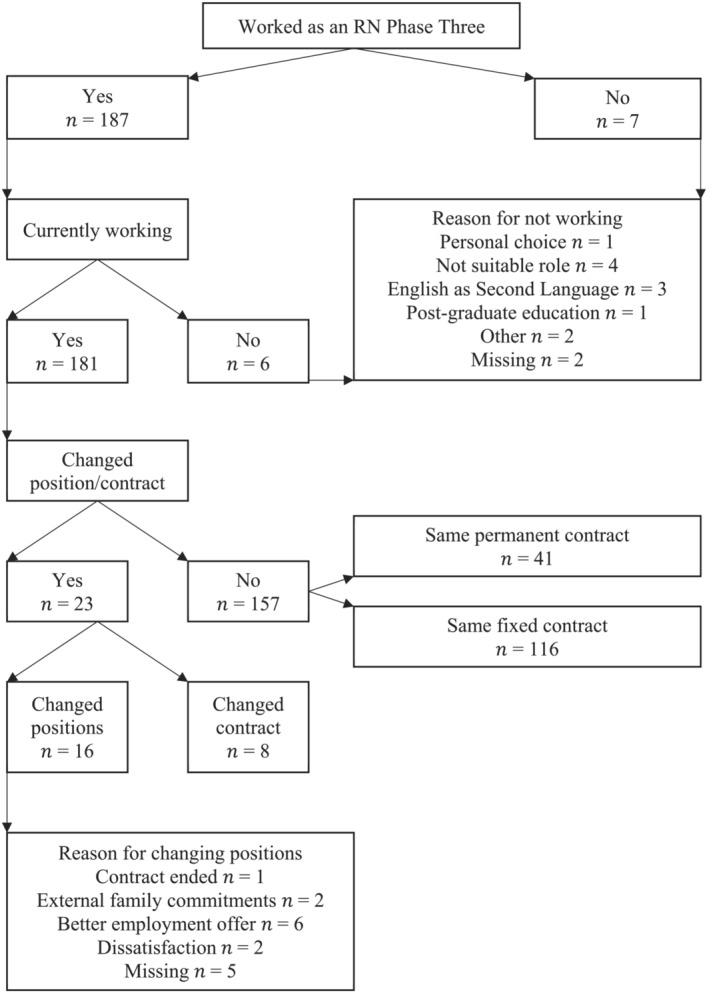
Flow of participants' employment outcomes at Phase Three

Thirty‐three respondents (17%) were not working as registered nurses at Phase Two (Figure 1), which decreased to 13 (6.7%) by Phase Three (Figure 2). After 12 months, the turnover rate from a nursing position was 8.2% (*n* = 16). The most common reason for changing jobs was for a better nursing position. The turnover rate from the profession was 6.7% (*n* = 13), consisting of 3.1% (*n* = 6) leaving a registered nurse job and not taking another, plus 3.6% (*n* = 7) never beginning a registered nurse job post‐graduation.

### 3.3 Work environment

The Work Environment, Support and Encouragement scale has very good internal consistency (previous Cronbach's alpha coefficient reported .92; Buffington et al., 2012; current study .86). Total scores range from 30 to 120, with higher scores suggesting stronger work environments with better support and encouragement. The overall average scores (Phase Two = 90.98, Phase Three = 90.21) suggest that most respondents agreed that their work environment was providing encouragement and support.

### 3.4 Support

**FIGURE 3:**
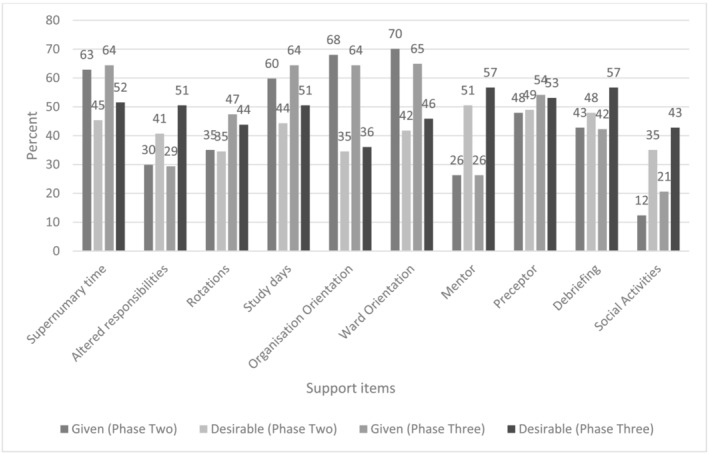
Support items provided and desired as percentage of the total sample

Most respondents identified as taking part in a formal structured support programme (79.9%). Many of these programmes lasted 12 months (74.2%). Participants were asked whether common support items were provided to them by employers and whether they identified the same support items as desirable in assisting their transition. Results are shown in Figure 3 as frequencies. Supernumerary time, study days, and orientation to both the organisation and ward were common support strategies implemented by employers, whereas altered responsibilities, mentors, and social activities were commonly requested by respondents. As time went on, there was an increase in all support items that respondents identified as desirable, with the biggest increase seen in wanting altered responsibilities. However, except for ward rotations and social activities, most support items given by employers remained relatively stable or decreased.

### 3.5 Stress

Most respondents (Phase Two *n* = 121; Phase Three *n* = 115) did not report stress from their personal lives that impacted on their work. However, for those that did, the majority noted that it had a negative impact on their work. The most common causes of this stress were personal relationships and finances.

### 3.6 Job satisfaction

Participants were asked the open‐ended question “What keeps you working in your current job?” Analysis found three themes: Supportive team (Phase One n = 63, Phase Two n = 72), job satisfaction (Phase One n = 69, Phase Two n = 70) and completing a formal structured support programme (Phase One n = 49, Phase Two n = 52). The most common response was the importance of having a supportive team. Participant 140 wrote, “what keeps me going is the support I'm getting from other nurses on the ward, they have been so lovely and great to me as a grad and has definitely been the biggest factor is making the transition from student to nurse easy.”

Job Satisfaction scale showed good internal consistency (Cronbach's alpha coefficient .85). Higher total scores (range 13–65) indicating higher job satisfaction. The average score for Phase Two was 49.04, and 46.23 for Phase Three indicating that most respondents were between “neither satisfied or dissatisfied” and “moderately satisfied” with their jobs on the 5‐point Likert scale.

### 3.7 Intention to remain

In Phases Two and Three, those who were working were asked how many more years they intended to remain in the nursing workforce in Australia, and in Phase Three only, how long they planned to remain in their current registered nurse position. As seen in Table 1, there was a drop in average years intended to remain in the workforce of 3.6 years, and a large difference between intention to remain in the workforce and intention to remain in a current position of 15.34 years. The Wilcoxon signed rank test revealed a statistically significant reduction in years intended to remain in the registered nurse workforce in Australia, *z* = −1.95, *p* = .05, with a small effect size (*r* = .11). The median score in years intended to remain in the registered nurse workforce in Australia decreased from Phase Two (Md 30) to Phase Three (Md 20).

**TABLE 1:** Intention to remain in employment descriptive statistics

	n	Range	Mean	SD	Kolmogorov–Smirnov^a^		
					Statistic	df	Sig.
Phase two							
Workforce	155	0–65	26.82	16.81	0.95	155	<.001
Phase three							
Workforce	173	0–65	23.22	16.80	0.92	173	<.001
Current position	173	0–65	7.88	13.31	0.57	173	<.001

*Note*: df = degrees of freedom; SD = standard deviation.

^a^ Lilliefors significance correction.

As shown in Table 2, most respondents in Phase Two (88.7%) and Phase Three (79.4%) were satisfied with nursing as a career. However, by Phase Three, only half of the respondents wanted to remain in their current position for the next 5 years. Similarly, approximately half felt they had been in their position “as long as they wanted to be.”

**TABLE 2:** Level of agreement with intention to remain items

Item	Strongly disagree/disagree	Agree/strongly agree	Missing
	n (%)	n (%)	n (%)
16 P2	15 (7.7)	172 (88.7)	7 (3.6)
16 P3	21 (10.8)	154 (79.4)	19 (9.8)
32 P2	116 (59.8)	69 (35.6)	9 (4.6)
32 P3	108 (55.7)	68 (35.1)	18 (9.3)
33 P2	150 (77.3)	36 (18.5)	8 (4.1)
33 P3	141 (72.7)	36 (18.5)	17 (8.8)
46 P2	66 (34.0)	121 (62.4)	7 (3.6)
46 P3	84 (43.3)	92 (47.4)	18 (9.3)

*Note*: 16 = I am satisfied with choosing nursing as a career; P2 = Phase Two; P3 = Phase Three; 32 = I have been in my position about as long as I want to be; 33 = If the economy was better I would think about finding another job; 46 = I would like to be working here in 5 years' time.

In Phases Two and Three, participants were asked: “What might cause you to leave your current place of employment?” In Phase Two, there were 152 responses, and 169 in Phase Three. In both phases, the same themes were identified, most commonly “dissatisfaction” (Phase Two *n* = 56, Phase Three *n* = 61), and “better opportunities” (Phase Two *n* = 59, Phase Three *n* = 73). Many responses in both phases centred on leaving their current employment for better opportunities. Participant 145 wrote, “Better career advancement offer or like an offer to work in the hospital where I want to be.”

In Phases Two and Three, all participants were asked: “What do you think could be done to improve Registered Nurse retention in the Australian workforce?” In Phase Two, there were 160 responses, and 165 in Phase Three. Across the two phases, three themes emerged: Improved working conditions (Phase Two *n* = 71, Phase Three *n* = 68), increased support and recognition (Phase Two *n* = 68, Phase Three *n* = 57), and more employment opportunities (Phase Two *n* = 27, Phase Three *n* = 24). The most reported theme was an improvement in working conditions, specifically patient‐to‐nurse ratios, remuneration, and shift options. Participant 146 wrote, “Better/consistent nursing ratios (often nurses are pressured to accept patient ratios that fail to conform to current Australian standards), increased pay with reference to the increased cost of living, flexible working hours.”

Which factors influence early career nurse intention to remain in the profession?

Direct binary logistic regression was performed to identify which factors influenced intention to remain in the profession longer than 10 years (see Table 3). The full model containing six predictors was statistically significant, *X* ^2^ (6, n = 131) = 14.02, p = .029, thus was able to distinguish between respondents who intended to remain in the profession longer than 10 years and those who did not. Work Environment, Support and Encouragement score [Phase Two] was the only significant variable; for every point, early career nurses scored higher on the Work Environment, Support and Encouragement scale; they were 1.07 times more likely to report intending to remain for more than 10 years.

**TABLE 3:** Binary logistic regression modelling to predict intention to remain in the profession

Variable	B	SE	OR	95.0% CI	Wald	p
Phase two						
Grade point average	−0.98	0.54	0.38	[0.13–1.08]	3.28	.070
Undergraduate employment	0.51	0.63	1.66	[0.49–5.66]	0.66	.416
Health care experience	−0.34	0.47	0.71	[0.28–1.80]	0.52	.473
Stress P2	−0.24	0.49	0.78	[0.30–2.61]	0.24	.621
Job satisfaction P2	−0.04	0.04	0.96	[0.89–1.04]	1.13	.287
Work environment, support and encouragement S P2	0.06	0.03	1.07	[1.01–1.12]	5.65	.017
Phase three						
Age	0.41	0.48	1.51	[0.59–3.84]	0.74	.239
English as a second language	0.98	0.54	2.66	[0.93–7.66]	3.29	.070
Health care experience	−0.55	0.44	0.58	[0.25–1.35]	1.62	.203
Stress P3	1.08	0.49	2.95	[1.14–7.63]	4.95	.026
Job satisfaction P3	−0.02	0.05	0.99	[0.92–1.05]	0.20	.653
Readiness for practice scale P1	−0.08	0.05	0.92	[0.84–1.02]	1.64	.104
Readiness for practice scale P2	0.03	0.06	1.03	[0.91–1.16]	0.19	.659
Readiness for practice scale P3	0.05	0.06	1.05	[0.93–1.18]	0.64	.425
Work environment, support and encouragement P2	0.01	0.02	1.01	[0.97–1.06]	0.17	.676
Work environment, support and encouragement P3	0.05	0.03	1.05	[1.00–1.11]	3.81	.050

*Note*: CI = confidence interval for odds ratio (OR); P2 = Phase Two; P1 = Phase One; P3 = Phase Three.

In the Phase Three, the full model containing 10 predictors was also statistically significant, *X* ^2^ (10, n = 131) = 21.51, p = .018. Higher Work Environment, Support and Encouragement scores remained predictive of wanting to remain in the profession; however, the strongest predictor was having stress. Those with stress were three times more likely to remain in the profession than those without.

Which factors influence early career nurse intention to remain in their current position?

Direct logistic regression was performed to identify the factors that impact on intention to remain in their current position longer than 7.5 years (see Table 4). The full model containing 12 predictors was not statistically significant, *X* ^2^ (12, n = 148) = 17.15, p = .114.

**TABLE 4:** Binary logistic regression modelling to predict intention to remain in current position

Variable	B	SE	OR	95.0% CI	Wald	p
Age	0.65	0.48	1.92	[0.76–4.87]	1.88	.170
Grade point average	−0.02	0.50	0.98	[0.37–2.62]	0.00	.973
English second language	−0.30	0.61	0.74	[0.23–2.43]	0.25	.619
Undergraduate employment	−1.30	0.61	0.27	[0.08–0.90]	4.52	.033
Health care experience	−0.18	0.53	0.83	[0.30–2.34]	0.12	.730
Stress (P3)	0.10	0.51	1.11	[0.41–3.00]	0.4	.840
Job satisfaction (P3)	0.04	0.04	1.04	[0.97–1.12]	1.28	.257
Readiness for practice scale P1	−0.04	0.05	0.96	[0.88–1.06]	0.70	.401
Readiness for practice scale P2	−0.80	0.06	0.92	[0.82–1.04]	1.80	.180
Readiness for practice scale P3	0.02	0.06	1.02	[0.90–1.15]	0.09	.767
Work environment, support and encouragement P2	0.01	0.02	1.01	[0.97–1.06]	0.26	.610
Work environment, support and encouragement P3	0.03	0.3	1.03	[0.98–1.09]	1.28	.258

*Note*: CI = confidence interval for odds ratio (OR); P2 = Phase Two; P1 = Phase One; P3 = Phase Three.

## 4 DISCUSSION

A key finding from the current study is that while most respondents were satisfied with nursing as a career and their workplace, half felt they had been in their position as long as they wanted to be, and only half wanted to remain in their current position for the next 5 years. Concurrently, a large difference between intention to remain in the workforce and the intention to remain in a current position was also found. This suggests that the early career nurses were satisfied with their career choice and intend to remain in the profession but also wanted to move positions early in their career, reflecting wider research (Brotherhood of St Laurence, 2017; Kidd et al., 2014; Wilkins & Inga, 2018). This desire for job mobility may reflect the younger demographic. In general, younger workers are more likely to move in and out of education, change careers, up‐skill and move location, all which impact on their employment (Fell, 2018). For nurses, it has historically been argued that job mobility is due to the female domination of the workforce, who historically have taken on more parenting and/or home duties (Health Workforce Australia, 2014). However, in wider research, job mobility is seen as a crucial mechanism through which superior employment outcomes, such as work–life balance and remuneration, are realised (Kidd et al., 2014).

In the past, dissatisfaction with the workplace has dominated the discussion on position turnover (Bontrager et al., 2016; Kenny et al., 2016). However, this study showed that most respondents were satisfied with the workplace, but only half wanted to remain in their current position for the next 5 years. The most common reason given for changing or intending to change employers was for a better employment offer. The open‐ended responses gave an insight into what this “better” employment may look like. Respondents reported looking for career advancement with opportunities to increase skills and searching for stability through permanent contracts with increased pay and flexible hours. Therefore, it can be suggested that like wider research (Kidd et al., 2014), job mobility in the early career nurse population is also for labour market gains such as work–life balance and remuneration. If an administrator only looks at turnover from a single nursing unit or even facility in isolation, that could be perceived negatively without these other pieces of information, which suggest nurse managers should be supported if they are encouraging career development opportunities. To retain early career nurses in a position in the future, employers may need to invest in initiatives such as increasing opportunities for continuing professional development, facilitating flexible career pathways, including the transfer between departments, organisations, and sectors, and flexible working contracts, to make staying in a position more attractive (House of Commons, 2016).

In this study, higher work environment support and encouragement scores were predictive of intention to remain in the profession. This finding is like that of previous research that showed that positive work environments retain early career nurses (Kramer et al., 2012). Conversely, negative perceptions of the work environment are strong predictors of intent to leave a position (Kramer et al., 2012; Lee et al., 2019) and the profession (Guerrero et al., 2017). Many studies have shown this positive correlation using a variety of tools. What this study adds is the comparison of intent to leave the profession and a position simultaneously using longitudinal follow‐up in a cohort of early career nurses from more than one institution and employed in various settings. Employers should understand that positive work environments increase an early career nurse's intention to remain in the profession, but this will not necessarily result in an increased intention to remain in their current position. Despite this, it is still important for workplaces to invest in creating positive work environments as they improve quality of care (Purdy et al., 2010) and minimise transition shock (Kim & Yeo, 2019), whereas negative perceptions will still encourage early career nurses to leave (Guerrero et al., 2017). However, with the knowledge that job mobility is common for early career nurses, future research should focus on ensuring early career nurse retention in the profession, not a position; therefore, future studies will require multiple sites and longitudinal follow‐up.

In this study, having stress in an early career nurse's personal life was predictive of intention to remain in the profession. This is a point of interest as there may be a perception that the reverse would be true. In the current study, those with stress were three times more likely to remain in the profession than those without. This contrasts with stress caused by the workplace, which has been a predictor of intent to leave a position (Wu et al., 2012; Yeh & Yu, 2009). This finding is interesting and may be explained by the identified causes of this stress: Personal relationships and finances. Historically, it was thought that retention of early career nurses could be improved by providing good working conditions such as removing obstacles to caregiving, allowing autonomy, and reducing workloads and other job pressures (Li et al., 2014). But generational changes may augment the focus of stress‐related research. Millennials foster strong social connections with friends and family (Lavoie‐Tremblay et al., 2008). Recognition of the need for early career nurses to develop work–life balance quickly during their transition to practice, and facilitation of this by employers, may alleviate some causes of stress for early career nurses that impact on their work. Future research needs to compare work‐related stress and personal stress to maintain the distinction between work elements that require change and personal circumstances of early career nurses that require support to overcome.

### 4.1 Limitations

The question pertaining to intent to remain in position was only included in the final survey thus prohibiting the ability to measure a change in intent to remain in the position over time. The participants were from two metropolitan institutions in two states in Australia and this may limit generalizability of results to the wider graduate populations. Furthermore, although self‐report measures are widely used, results may be affected by context, limits in memory, and social desirability bias. This research used a variety of strategies to mitigate these effects, such as assuring participants of their anonymity and their right to refuse to answer questions.

## 5 CONCLUSION

There are differences, in length and factors, when comparing early career nurses' intention to remain in their position and the profession. Early career nurses in this study were satisfied with their career choice and intended to remain in the profession but also intended to move positions early in their career. This study suggests turnover from a position may be a consequence of the desire for career progression for early career nurses within the profession.

## 6 IMPLICATIONS FOR NURSING MANAGEMENT

Previously, the focus of early career nurse turnover has been focussed on dissatisfying work environments. Whilst this study shows positive work environments, combined with appropriate supports and remuneration, remain imperative to retaining early career nurses in the profession, it also highlights that many early career nurses may want to change positions irrespective of satisfaction with current employment. Therefore, nurse managers should be supported to encourage career development and progression opportunities for early career nurses to ensure retention in the profession as opposed to retention in a position.

## CONFLICT OF INTEREST

The authors have no potential sources of conflict of interest to disclose.

## ETHICS STATEMENT

Ethical clearance was obtained from the UQ Behavioural & Social Sciences Ethical Review Committee (BSSERC), clearance number: NMSW 2016/02.

## Data Availability

The data that support the findings of this study are available on request from the corresponding author. The data are not publicly available due to privacy or ethical restrictions.
